# Density Scaling of Translational and Rotational Molecular Dynamics in a Simple Ellipsoidal Model near the Glass Transition

**DOI:** 10.3390/ijms23094546

**Published:** 2022-04-20

**Authors:** Karol Liszka, Andrzej Grzybowski, Kajetan Koperwas, Marian Paluch

**Affiliations:** 1 Institute of Physics, University of Silesia in Katowice, ul. 75 Pulku Piechoty 1, 41-500 Chorzow, Poland; karol.liszka@us.edu.pl (K.L.); kajetan.koperwas@us.edu.pl (K.K.); marian.paluch@us.edu.pl (M.P.); 2 Silesian Center for Education and Interdisciplinary Research, ul. 75 Pulku Piechoty 1a, 41-500 Chorzow, Poland

**Keywords:** density scaling, molecular anisotropy, glass transition, supercooled liquids, Gay–Berne model, molecular dynamics simulations

## Abstract

In this paper, we show that a simple anisotropic model of supercooled liquid properly reflects some density scaling properties observed for experimental data, contrary to many previous results obtained from isotropic models. We employ a well-known Gay–Berne model earlier parametrized to achieve a supercooling and glass transition at zero pressure to find the point of glass transition and explore volumetric and dynamic properties in the supercooled liquid state at elevated pressure. We focus on dynamic scaling properties of the anisotropic model of supercooled liquid to gain a better insight into the grounds for the density scaling idea that bears hallmarks of universality, as follows from plenty of experimental data collected near the glass transition for different dynamic quantities. As a result, the most appropriate values of the scaling exponent γ are established as invariants for a given anisotropy aspect ratio to successfully scale both the translational and rotational relaxation times considered as single variable functions of density^γ^/temperature. These scaling exponent values are determined based on the density scaling criterion and differ from those obtained in other ways, such as the virial–potential energy correlation and the equation of state derived from the effective short-range intermolecular potential, which is qualitatively in accordance with the results yielded from experimental data analyses. Our findings strongly suggest that there is a deep need to employ anisotropic models in the study of glass transition and supercooled liquids instead of the isotropic ones very commonly exploited in molecular dynamics simulations of supercooled liquids over the last decades.

## 1. Introduction

A rapid slowdown in molecular dynamics of supercooled liquids approaching the glass transition has been intensively studied since the 1960s and still attracts a lot of research interest as a phenomenon strongly related to the glass transition, the commonly accepted theory of which is continuously sought after. Initially, due to measurement limitations, the experimental study of molecular dynamics timescale or other dynamic quantities such as viscosity or diffusivity was conducted mostly as a function of temperature at ambient pressure. Consequently, the theoretical models that attempted to explain the mystery of supercooled liquid dynamics did not consider the effect of pressure on the molecular dynamics near the glass transition. In this context, the original version of the prominent Adam–Gibbs model [1] should be mentioned.

A development of high-pressure measurement techniques enabled researchers to investigate molecular dynamics of supercooled liquids and the glass transition at elevated pressure. A few decades’ worth of high-pressure measurements performed typically in isobaric or isothermal conditions has shed new light on the properties of molecular dynamics near the glass transition [2]. The main outcome of these research efforts was a strong suggestion that the contemporary models of the glass transition and related phenomena should not neglect the pressure effect on molecular dynamics [2,3]. To respond to this challenge, several models have been formulated as functions of temperature *T* and pressure *p* [4,5,6,7]. However, it seems that the most promising approach is the density scaling idea also known as thermodynamic scaling, which has been widely explored since the beginning of the 21st century [2,3,8,9,10]. There are two crucial advantages of this idea over various other models: (i) An expected relation between macroscopic quantities determined from experimental data with intermolecular interactions that govern molecular dynamics at least in highly viscous systems, especially including supercooled liquids. (ii) Firm evidence of density scaling behavior demonstrated by experimental data analyses.

The link between the macroscopic phenomena and their underlying molecular mechanisms has been suggested via the exponent *γ*, which enables the scaling of various dynamic quantities measured in different thermodynamic conditions onto a single master curve according to the power density scaling (PDS) law that can be expressed for structural relaxation times *τ* as follows:

(1) $$\tau =f\left(\rho^{\gamma }/T\right),$$

where the scaling function argument involves thermodynamic variables, the temperature *T* and the density *ρ* dependent in general on *T* and *p*, respectively, as well as the scaling exponent *γ* established as a material constant independent of thermodynamic conditions in a vast majority of known cases tested experimentally [2,3,10]. The PDS law has been validated by using measurement data collected mainly in the supercooled liquid state of many materials that belong to van der Waals liquids and ionic liquids, but also for polymer melts, and even oils [11,12] and some liquid crystal phases [13,14]. Thus, there are numerous experimental cases confirming the PDS law for different dynamic quantities such as viscosity, dc conductivity (in ionic liquids), structural relaxation time, segmental and chain relaxation time (in polymers), and even timescales of some other relaxation processes usually unrelated to the glass transition. On the other hand, it has been strongly suggested that the scaling exponent *γ* is related to an effective short-range intermolecular potential relevant to molecular dynamics of viscous systems, which is composed of a weak attractive term and a dominant repulsive term given by an inverse power law (IPL), $U_{IPL}∼r^{-3\gamma }$, where *r* is an intermolecular distance [15,16,17,18,19,20,21,22]. 

The suggested important role of the scaling exponent *γ* aroused a lot of research interest aimed at working out methods for determining the scaling exponent value for a given material and better understanding its relation to the rules that govern molecular dynamics near the glass transition. Many observations of the scaling behavior of experimental data have led to the formulation of the density scaling criterion [23]:

(2) $$\log_{10}T=\gamma \log_{10}\rho +C_{\tau }\;\mathrm{at}\;\tau =const,$$

where *C_τ_* is a constant dependent on *τ*. This criterion considered in the entire experimental range constitutes a fundamental equation equivalent to the PDS law and provides a very useful method for finding the scaling exponent values, which enables us to establish the scaling exponent *γ* without applying auxiliary models such as the temperature–density versions of the Avramov and MYEGA models formulated in the density scaling regime. Consequently, one can investigate the density scaling properties in a way that is not burdened with specific assumptions going beyond the density scaling idea, which have been additionally made to construct the mentioned models of the thermodynamic evolution of dynamic quantities. On the other hand, extensive study of the theoretical grounds for the density scaling has been attempted by using molecular dynamics (MD) simulations based mainly on the isotropic models of supercooled liquids, which only involve the commonly known Lennard–Jones (LJ) potential, such as the Kob–Anderssen binary LJ [24] and Wanström binary LJ liquids [25]. As a result, the theory of isomorphs has been formulated [26], which initially seemed to provide solid theoretical fundamentals for the density scaling of dynamic quantities determined from experimental data. However, a further development of the theory of isomorphs, which was accompanied with the extended simulation investigations still carried out mostly in isotropic models [27,28], has shown that there are some doubts about the theoretical significance for the experimental data analyses that are satisfactorily performed in terms of the PDS law. One of the conclusions drawn from the initial stage of works on the theory of isomorphs is the linear virial–potential energy (WU) correlation between the instantaneous values of the total system virial <W> and the total system potential energy <U>, where the brackets < > mean the ensemble average. The WU correlation was expected at least in isochoric conditions, and invoking the effective intermolecular potential based on the IPL repulsive term, it was supposed to yield the scaling exponent value as a slope coefficient of this correlation [18,19,29]. Besides the theory of isomorphs, there was another theoretical attempt made in a phenomenological manner to formulate a uniform description of dynamic and thermodynamic properties near the glass transition based on the density scaling idea. According to the approach, the scaling exponent value could be evaluated as a fitting parameter of some equation of state (EoS) based on the assumption of the effective potential of the dominant IPL repulsive term for supercooled liquids [30,31,32,33,34].

A main motivation of the paper is a considerable discrepancy between the values of the exponents *γ* and *γ_EoS_* commonly obtained from experimental data analyses, which can satisfy the PDS law given by Equation (1) and can be found by the fitting pressure–volume–temperature (*pVT*) data to the EoS given by Equation (3), respectively [2,10]. The analogous analyses based on the data collected from MD simulations in simple isotropic models, however, yield an equality of *γ* and *γ_EoS_* to a good approximation, which well corresponds with the WU correlation results [33,34]. 

In this paper, we investigate the effect of anisotropy reflected in both the molecular shape and the intermolecular potential on the density scaling properties of the Gay–Berne (GB) model [35] well known in the computer simulations of liquid crystals, but also validated to study the supercooled liquid state and the glass transition at zero pressure by Kapko and Angell [36]. We perform MD simulations in this model in a wider thermodynamic range to explore the supercooled liquid state and the glass transition achieved at both zero and elevated pressure. Considering several anisotropy conditions well defined in the GB model, we thoroughly analyze the obtained volumetric data as well as the translational and rotational molecular dynamics to gain a better insight into the fundamentals for the density scaling idea.

## 2. Results and Discussion

### 2.1. Volumetric Data Analysis

Although our main interest in the *pVT* data collected from the NPT MD simulations performed in the GB model, which are described in detail in Section 3, concerns the density scaling properties, we first analyze the data to identify the glass transition curve. This is a very important task, because it is not easy to achieve the supercooling state for the exploited GB model due to its high tendency to crystallize, which has been useful to model liquid crystal phases. Kapko and Angell [36] established that it was possible to achieve the supercooling liquid state and the glass transition at zero pressure for an anisotropy aspect ratio *α_r_* near 1.4 by using their parametrization in the GB model. We tested the GB systems of the aspect ratio between 1.2 and 1.6 in the pressure range between 0 and 20 and the temperature range between 0.1 and 2.0 in LJ units. Taking into account the occurrence of the glass transition and the typical behavior of the isobaric dependences of volume on temperature in the supercooled liquid state, we decided to limit our further simulation study to the aspect ratios, $1.3\leq \alpha_{r}\leq 1.45$, and the thermodynamic range, 0.1≤T≤1.0 and 0≤p≤5.0. In the selected simulation range, neither translational nor rotational liquid crystal ordering has been detected, which has been confirmed by the obtained values of the order parameter *S_2_* and the analysis of the radial distribution functions and rotational radial distribution functions as described in Section 3. 

**Figure 1:**
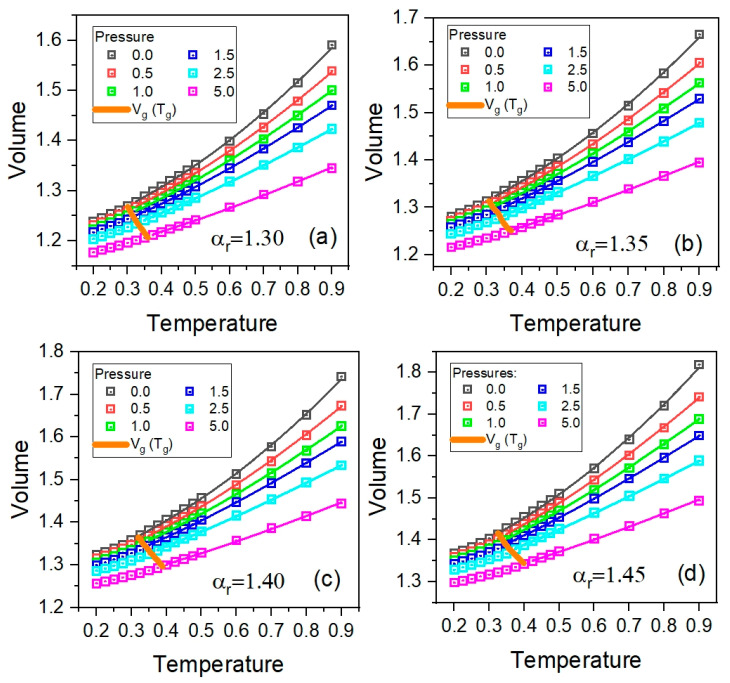
The plots of the isobaric dependences of the particle number volume *V* on temperature *T* in the glassy and supercooled liquids states in the GB model, which are presented in the panels for the different anisotropy aspect ratios *α_r_*: (**a**) 1.30, (**b**) 1.35, (**c**) 1.40, and (**d**) 1.45, respectively. The solid curves crossing the isobaric dependences *V* on *T* denote the glass transition curves and the solid curves along the dependences *V*(*T*) present the fitting curves to the EoS given by Equation (3) with the values of its fitting parameters collected in Table 1.

**Figure 2:**
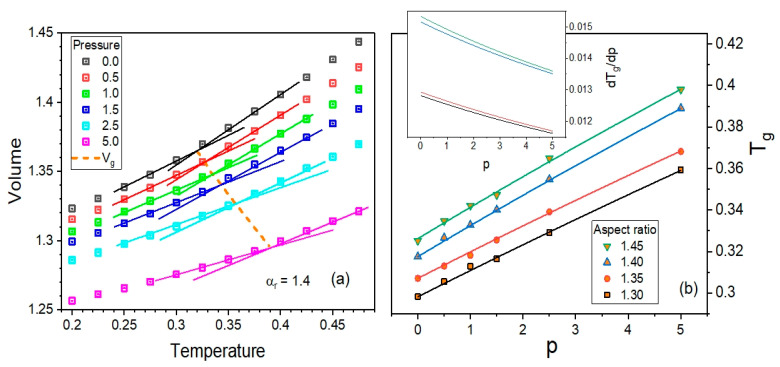
(**a**) Presentation of the method for determining the glass transition curve based on the *pVT* data for an aspect ratio equal to 1.40. (**b**) Plot of the dependences of the glass transition temperature *T_g_* on pressure *p* for the examined anisotropy aspect ratios *α_r_*. The solid curves represent fits to the Andersson–Andersson equation [37], and their derivatives calculated numerically are shown in the inset.

In the aforementioned thermodynamic range, we thoroughly investigated four GB systems of different anisotropy aspect ratios, *α_r_* = 1.30, 1.35, 1.40, and 1.45, carrying out the NPT MD simulations at pressures, *p* = 0, 0.5, 1.0, 1.5, 2.5, and 5.0. The *pVT* data collected from these simulations are presented in Figure 1, where the volume *V* denotes an inverse of the particle number density, that is, the average simulation box volume divided by the number of particles in the box. Moreover, in Figure 2a, an example of the method for determining the glass transition curve based of the *pVT* data for the aspect ratio *α_r_* = 1.40 is shown. 

For all examined anisotropy aspect ratios, one can reliably identify the glass transition curves depicted by dashed lines in the panels of Figure 1. Since the glass transition *T*-*p* curve is an important characteristic of glass-forming liquids, the dependences of the glass transition temperature *T_g_* on pressure *p* are additionally presented in Figure 2 for comparison. One can see that the dependences *T_g_*(*p*) increase with increasing *p*, which is a typical behavior known in most glass-forming materials. Moreover, the glass transition curve shifts to higher temperatures by increasing the anisotropy aspect ratio, which is a result that is worth considering in the experimental study of glass formers. 

Based on the fitting curves of the dependences *T_g_* on *p* to the Andersson–Andersson equation [37] commonly exploited to interpolate the experimental dependences *T_g_*(*p*) for many decades, it has also been possible to calculate numerically the pressure coefficient of the glass transition temperature, which is the derivative *dT_g_*/*dp* that constitutes a key parameter of the Ehrenfest equations [38,39]. As can be seen in the inset to Figure 2b, the pressure coefficient of the glass transition temperature increases when increasing the anisotropy aspect ratio, and decreases with increasing pressure for a given anisotropy. The latter well corresponds to the earlier results obtained from experimental data analyses [40], while the former is the next issue worthy of consideration in the experimental investigations of glass formers.

After the determination of the pressure dependences of the glass transition temperature, we were able to carry out a volumetric data analysis limited to the supercooled liquid state to which one can apply the following equation of state, well interpreted in terms of the density scaling fundamentals [32]:

(3) $$V(T,p)=V(T,p_{0}){\left[1+\frac{\gamma_{EOS}}{B_{T}(p_{0})}\left(p-p_{0}\right)\right]}^{1/\gamma_{EOS}},$$

where the exponent *γ_EoS_* is a fitting parameter. Additionally, in Equation (3), the functions of temperature parametrization for volume and isothermal bulk modulus at the reference state (*T*,*p*_0_) are given respectively as follows:

$$V(T,p_{0})={\left[\sum_{l=0}^{l=2}A_{l}{\left(T-T_{0}\right)}^{l}\right]}^{-1},$$

$$B_{T}(p_{0})=b_{0}\exp\left[-b_{1}(T-T_{0})\right],$$

where $b_{0}=B_{T_{0}}(p_{0})$, $b_{1}=b_{1}(p_{0})=-{\partial \ln B_{T}(T,p_{0})/\partial T|}_{T=T_{0}}$, $A_{0}=\rho^{-1}(T_{0},p_{0})$, and $A_{l}={(1/l!)\partial^{l}\rho^{-1}(T,p_{0})/\partial T^{l}|}_{T=T_{0}}$ for *l* = 1, 2, are fitting parameters, and $\left(T_{0},p_{0}\right)$ is a fixed reference state point, which is usually chosen near the glass transition at ambient pressure. Herein, we have selected $\left(T_{0},p_{0}\right)$ at *p*_0_ = 0 and the glass transition temperatures, $T_{0}=T_{g}\left(p_{0}\right)$, established at 0.298, 0.307, 0.319, and 0.325 for the examined anisotropy aspect ratios *α_r_* = 1.30, 1.35, 1.40, and 1.45, respectively.

As a result, we obtained (see Figure 1) very good-quality *pVT* data fits to Equation (3); the values of its parameters for all examined anisotropy aspect ratios are collected in Table 1. 

It should be stressed that the EoS has been derived in accordance with Euler’s theorem for homogeneous functions on the assumption of the effective short-range effective intermolecular potential characterized by the dominant repulsive IPL term and a weak attractive background [30]. Hence, one might have expected that the repulsive potential term should have been as follows: $U_{IPL}∼r^{-3\gamma_{EoS}}$. However, all known analyses of volumetric measurement data of supercooled liquids by using the EoS or its isothermal precursor yielded a value of *γ_EoS_* for a given material usually two times greater than the value of *γ* which scaled dynamic quantities for the material in terms of the PDS law [30,32,34,41,42,43]. On the other hand, our earlier tests [33] performed by using the KABLJ simulation model and its version limited only to the repulsive IPL potential term (KABIPL) with different exponents clearly show that $\gamma_{EoS}\approx \gamma$. Additionally, the values of *γ_EoS_* and *γ* were consistent with the slope coefficients of the linear WU correlations established for the isotropic models. In the next section, we discuss this issue based on the data collected from our MD simulations reported herein for the GB anisotropic model. 

### 2.2. Translational Dynamics

The molecular dynamics relevant to the glass transition is believed to be mainly related to structural relaxation in the case of prototypical supercooled liquids that belong to van der Waals liquids. In MD simulations that rely on simple isotropic models involving the LJ potential, the structural relaxation timescale data are typically sourced from the analysis of the incoherent self-scattering (ISS) function *F_s_*, which actually reflects only translational relaxation dynamics due to no rotations in the isotropic systems that consist of unbound point particles. However, the GB model that consists of unbounded ellipsoidal particles provides a convenient opportunity to investigate separately both the translational and rotational dynamics, which is usually difficult to achieve by using experimental spectroscopic techniques. In this section, we focus on the study of the translational motions of the centers of mass of the ellipsoidal GB particles of different anisotropy aspect ratios. 

Taking into account our MD simulation carried out in cubic simulation boxes on the assumption of three-dimensional periodic boundary conditions as well as the explored supercooled liquid state characterized by neither liquid crystal nor crystal ordering, our simulation systems can be classified as isotropic ones. Hence, to determine the translational relaxation timescale of the unbounded ellipsoidal GB particles, one may apply a methodology that is analogous to that typically employed in finding the molecular dynamics timescale in the simulation models that consist of the unbounded point particles. Thus, we determine the translational relaxation time *τ* from the known formula, $F_{s}\left(\tau \right)=e^{-1}$, where the ISS function has been evaluated at the value of the wave vector, *k*, at which the first maximum of the static structure factor *S*(*k*) occurs. In Section 3, additional information about the computation of the time correlation functions *F_s_* is supplied.

**Figure 3:**
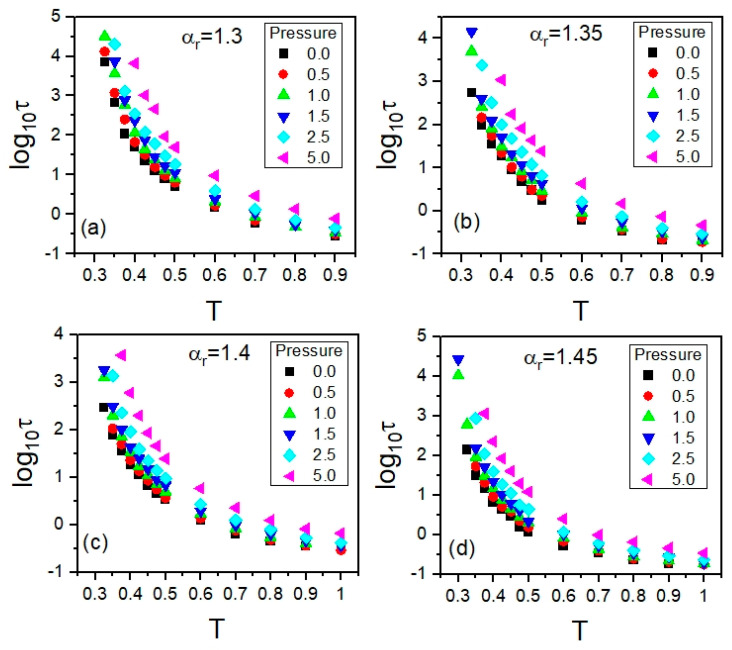
Plots of the isobaric dependences of translational relaxation times *τ* on temperature *T* in the supercooled liquid state in the GB model, which are presented in the panels for the different anisotropy aspect ratios *α_r_*: (**a**) 1.30, (**b**) 1.35, (**c**) 1.40, and (**d**) 1.45, respectively.

**Figure A1:**
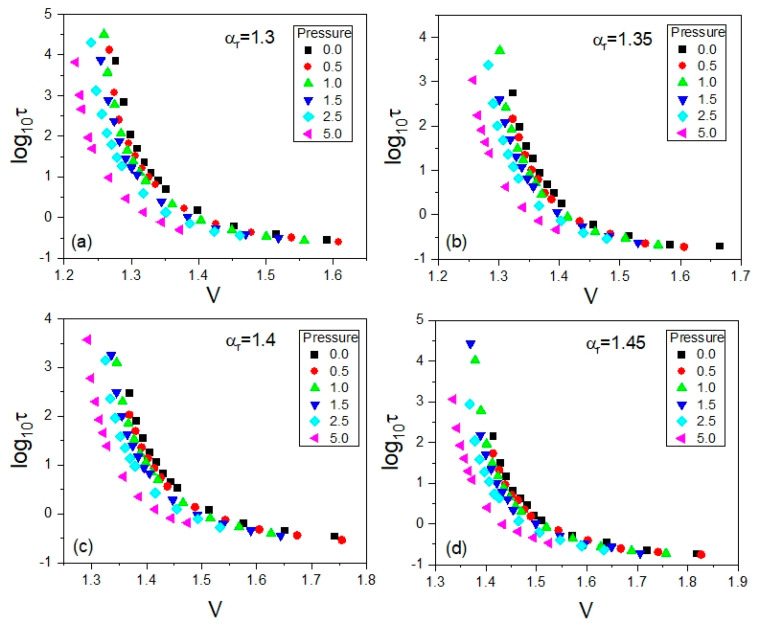
Plots of the isobaric dependences of translational relaxation times *τ* on the particle number volume *V* for different anisotropy aspect ratios α_r_ in the supercooled liquid state in the GB model. In the separate panels, there are the cases shown for the different anisotropy aspect ratios *α_r_*: (**a**) 1.30, (**b**) 1.35, (**c**) 1.40, and (**d**) 1.45, respectively.

From the aforementioned analysis of the ISS function at each state point (*T*,*p*) at which the NPT MD simulations were performed in the supercooled liquid state of the GB systems for all examined anisotropy aspect ratios, the translational relaxation times *τ* were determined. In this way, we obtained both the *T*-*p* and *T*-*V* dependences of *τ*, where the average values of the particle number volume *V* were found from the MD simulation in the NPT ensemble at each state point (*T*,*p*). From the NPT MD simulations carried out along selected isobars at *p* = 0, 0.5, 1.0, 1.5, 2.5, and 5.0, one can easily plot both the isobaric dependences τ(T) and τ(V). The former are shown in Figure 3, with a separate panel for each anisotropy aspect ratio. The corresponding isobaric dependences τ(V) are presented in Appendix A. It is worth mentioning that the dependences plotted in Figure 3 and Figure A1 well reflect the enormous slowdown in the molecular dynamics, which is characteristic of supercooled liquids approaching the glass transition.

As already noted, the data collected from the NPT MD simulations enable us to analyze the temperature–volume dependences of translational relaxation times established straightforwardly from the simulations. Thus, we can easily test the quality of the density scaling of the timescale of translational molecular dynamics in terms of the PDS law given by Equation (1), where *ρ* is the particle number density equal to the inverse of the particle number volume *V*, i.e., $\rho =V^{-1}$. 

**Figure 4:**
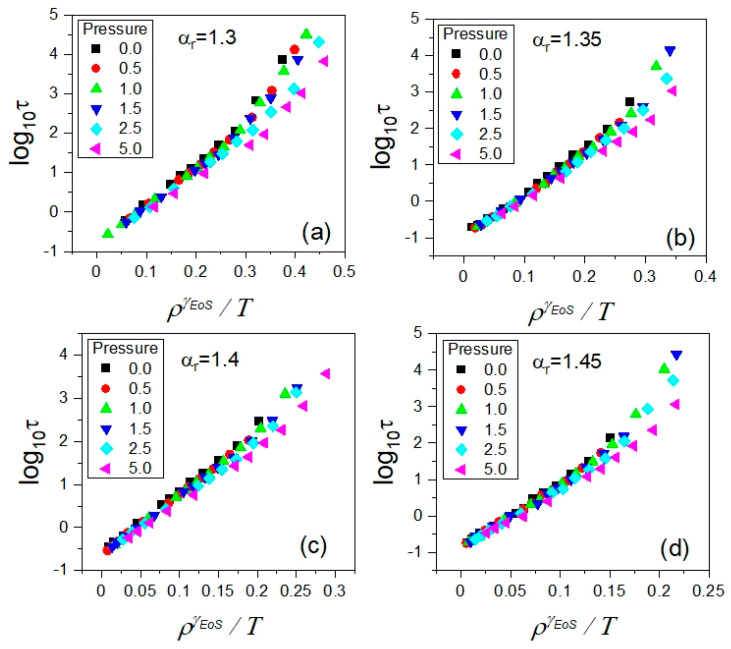
Plots of the attempts at employing the values of the density scaling *γ_EoS_* found as the fitting parameter of the EoS given by Equation (3) in the implementation of the density scaling of translational relaxation times *τ* collected in the supercooled liquid state in the GB model for all tested anisotropy aspect ratios *α_r_*, which are presented in the separate panels for different values of *α_r_*: (**a**) 1.30, (**b**) 1.35, (**c**) 1.40, and (**d**) 1.45, respectively. The values of *γ_EoS_* are collected in Table 1.

In our first test of the density scaling in the GB model considered in the supercooled liquid state, we assume that $\gamma \approx \gamma_{EoS}$ in Equation (1). Consequently, we observe that the scaling curves $\tau \left(\rho^{\gamma }/T\right)$ valid at short translational relaxation times considerably diverge when slowing down translational molecular dynamics as shown in Figure 4 for each anisotropy aspect ratio. This result differs from those obtained for the simulation data collected in the isotropic models of supercooled liquids such as the KABLJ and KABIPL models that consist of point particles and even in the simple three-point particle model of ortho-terphenyl (OTP), which enabled us to successfully scale dynamic quantities using the scaling exponent *γ_EoS_*. However, the experimental data measured for OTP in the supercooled liquid state have not yet provided such an opportunity [33].

Since the test of the density scaling represented in Figure 4 has failed, one could even suspect the invalidity of the density scaling of translational relaxation times in the supercooled liquid state in the GB model. To verify this issue, we used the fundamental formula that is the density scaling criterion. To apply Equation (2) to the dependences $\log_{10}\tau \left(T\right)$, we followed a similar procedure that has been well tested in many analyses of experimental data. We selected several values of the translational relaxation times *τ* that are represented in the explored thermodynamic range, and then we established the temperatures *T_τ_* at the crossing points of the isobaric temperature dependences of the translational relaxation timescale and the isochrones defined by *τ* = *const*. The corresponding densities $\rho_{\tau }(T_{\tau },p_{\tau })$ can be calculated from the EoS, for instance from Equation (3). In addition, to enhance the reliability of the isochronal dependences $\log_{10}T_{\tau }$ vs. $\log_{10}\rho_{\tau }$, the analysis can be supported by some interpolations of the dependences $\log_{10}\tau \left(T\right)$ usually determined insufficiently densely from measurements. Such evaluations not shown herein were also employed in our analysis of the isochronal temperature–density dependences $\rho =V^{-1}$.

**Figure 5:**
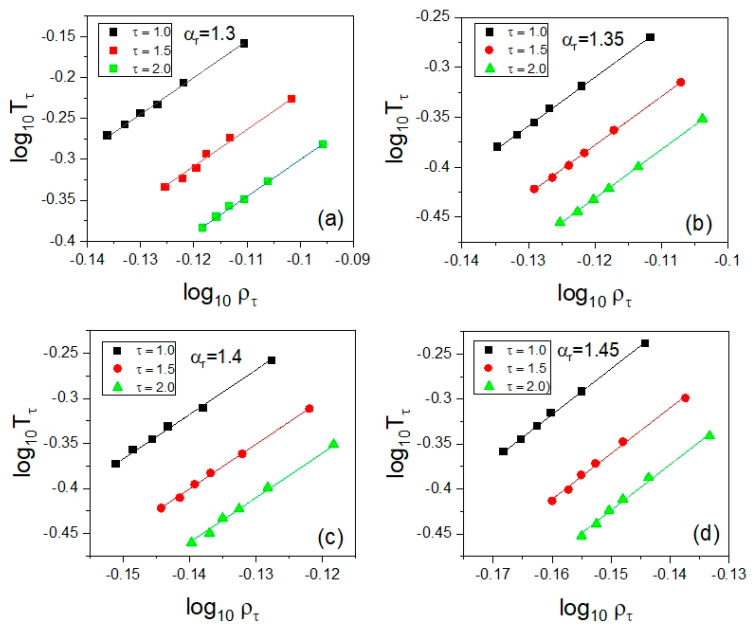
Presentation of the application of the density scaling criterion given by Equation (2) to the evaluation of the values of the scaling exponent *γ* based on the isochronal log–log dependences of *T* on *ρ* for each tested anisotropy aspect ratio *α_r_* in the GB model, which is shown in the separate panels for different values of *α_r_*: (**a**) 1.30, (**b**) 1.35, (**c**) 1.40, and (**d**) 1.45, respectively. The isochrones are determined at the selected constant values of the translational relaxation times *τ* in the supercooled liquid state.

**Figure 6:**
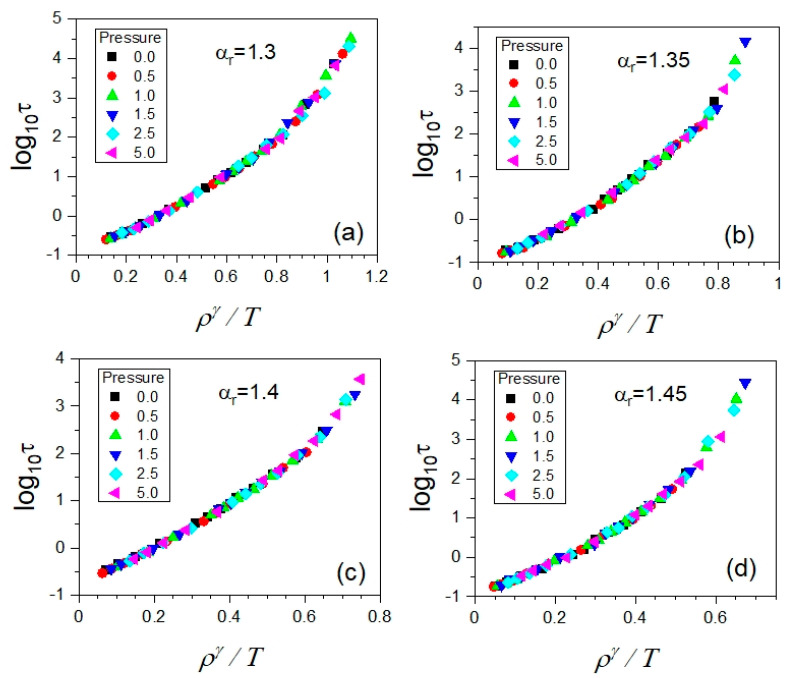
Plots of the density scaling of the translational relaxation times *τ* collected in the supercooled liquid state in the GB model, which is successfully carried out by using the values of the density scaling evaluated based on the density scaling criterion given by Equation (2). In the separate panels, there are the cases shown for the different anisotropy aspect ratios *α_r_*: (**a**) 1.30, (**b**) 1.35, (**c**) 1.40, and (**d**) 1.45, respectively.

As shown in Figure 5, we established the isochronal correlations between $\log_{10}T_{\tau }$ and $\log_{10}\rho_{\tau }$, finding their high-quality linear fits characterized by a single value of their slope coefficient *γ* for a given anisotropy aspect ratio (see Table 2). It means that the values of *γ* determined from the density scaling criterion applied to the translational relaxation times *τ* should lead to their density scaling according to a function $\tau \left(\rho^{\gamma }/T\right)$. Indeed, the expected outcome was successfully achieved, as presented in Figure 6, which constitutes the next example of the fundamental significance of the density scaling criterion to the PDS law.

In Appendix B, for the completeness of our comparative analysis of the density scaling ability, depending on the used scaling exponent *γ_EoS_* or *γ*, we also consider the values of *γ_EoS_* determined by fitting the simulation dependences $V\left(T,p^{conf}\right)$ to a precursor of Equation (3), which involves the configurational pressure *p^conf^* instead of the pressure *p*. Nevertheless, the analysis exploiting the other EoS (Equation (A1)) derived from the intermolecular potential dominated by the repulsive IPL term has not changed our conclusions that the molecular shape and intermolecular potential anisotropies that are characteristic of the GB model destroy the consistency between the values of *γ_EoS_* and *γ*. It gives evidence that the simple ellipsoidal GB model well reflects this inconsistency commonly observed from the experimental data analyses contrary to the isotropic simulation models most often used to study properties of supercooled liquids in the MD simulations. 

Previously, we also obtained such a discrepancy between the values of *γ_EoS_* and *γ* in MD simulations in the rhombus-like molecules (RLM) model introduced by us [44]. The RLM model enables one to study a mean effect of the molecular anisotropy on the examined macroscopic quantities, including the impact exerted by intramolecular forces. Nevertheless, the force field exploited in the RLM model is mainly based on the LJ and Coulomb potentials, which are classified as isotropic. Consequently, a quantification of the molecular anisotropy degree is complicated in the RLM model [44,45]. However, the GB model employed in the MD simulations performed herein is characterized by a well-defined measure of both molecular shape and potential anisotropy, because the molecular shape anisotropy determined via axes of ellipsoidal molecules is straightforwardly reflected in the parameters of the GB potential. Thus, the results of MD simulations performed earlier in the RLM model and here in the GB model give firm evidence that the discrepancy between the values of *γ_EoS_* and *γ* depends on the molecular anisotropy. For this reason, it cannot be reproduced in the manner obtained from measurement data if we exploit MD simulations in the simple isotropic models based on the LJ intermolecular potentials such as the KABLJ and three-point-particle OTP models. 

Since the density scaling of translational relaxation times has been very satisfactorily implemented in the supercooled liquid state in the anisotropic GB model, an interesting question arises as to whether the most effective values of the density scaling *γ* can also be deduced from the virial–potential energy correlation postulated in the initial version of the theory of isomorph [18,19,29]. This question is important, because the theory of isomorphs has had a lot of interest from researchers involved in the study of density scaling and has been widely discussed as providing the sought-after theoretical fundamentals for the density scaling idea. 

In the initial works on the isomorph theory, a linear correlation of the instantaneous values of the average system virial <W> and the average system potential energy <U> has been suggested for model systems of supercooled liquids as the reason for the density scaling of their molecular dynamics [17]. Nevertheless, the initial studies conducted within the framework of the isomorph theory have undoubtedly shown that the slope coefficient of the linear WU correlation depends on density, indicating an isochoric character of the linear WU correlation, the slope coefficient *γ_WU_* of which has been considered as the density scaling exponent [18,19,29,33]. The further development of the isomorph theory has suggested that the scaling exponent γ is state-point dependent, and even a few methods have been worked out for verifying the hypothesis based on the experimental data [46,47,48,49,50]. Although applications of the methods to analyze measurement data have indeed given evidence for the hypothesis of the state-point dependent scaling exponent, no one has achieved the density scaling of the dynamic quantities exploiting the state-point-dependent values of *γ* until recently. This problem definitely goes beyond our investigations reported herein, but it is worthy of mention that its solution most likely consists in finding a proper form of the scaling function, which would be different from the PDS law and enable one to utilize the state-point-dependent values of *γ* to scale the dynamic quantities. However, in this context, it should be stressed that even if we could meet this ambitious challenge in the future, the scaling exponent *γ* considered as a material constant independent of thermodynamic conditions would not be neglected in the analyses based on the density scaling idea. This is because it would still be an effective value of *γ* averaged to a very good approximation in the case of many materials, which could valuably provide information about the main features of molecular dynamics simulations near the glass transition. Additionally, it is worth mentioning on this point that despite a phenomenological character of the theory of isomorphs, its origin being based on the simulation data rather than the experimental one has been promising from a cognitive point of view. However, the simulations [17,18,19,22,26,27,28,29] underpinning the isomorph theory were mainly carried out in the isotropic models based on the LJ potential or its generalized version consisting of the repulsive and attractive parts characterized by various exponents different from those typically assumed to equal 12 and 6, respectively.

**Figure 7:**
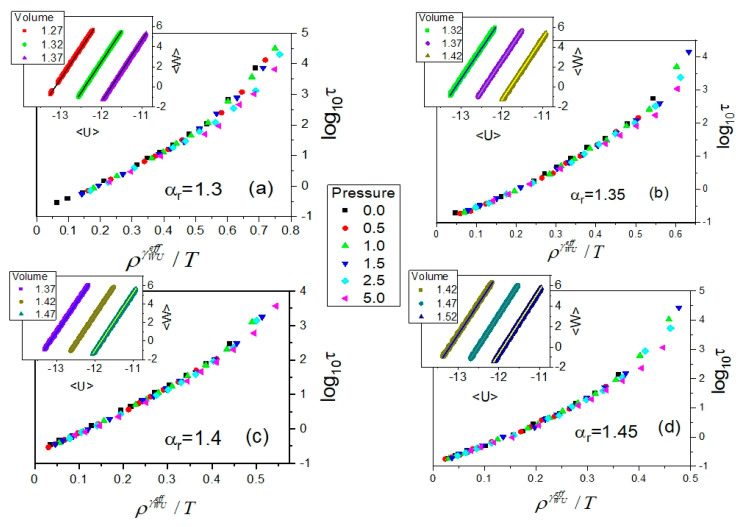
Plots of the attempts at employing the values of the density scaling $\gamma_{WU}^{eff}$ found from the analysis of the WU correlation as its slope coefficient in the implementation of the density scaling of translational relaxation times τ collected in the supercooled liquid state in the GB model. In the separate panels, there are the cases shown for the different anisotropy aspect ratios *α_r_*: (**a**) 1.30, (**b**) 1.35, (**c**) 1.40, and (**d**) 1.45, respectively. In the insets to the subsequent panels, the tested isochoric WU correlations are represented at volumes (1.27, 1.32, 1.37), (1.32, 1.37, 1.42), (1.37, 1.42, 1.47), and (1.42, 1.47, 1.52) for *α_r_* = 1.30, 1.35, 1.40, and 1.45, respectively. The estimated values of $\gamma_{WU}^{eff}$ used in the scaling plots are listed in Table 3.

For these reasons, we focus on the analysis of the WU correlation collected in the anisotropic GB supercooled liquid from the viewpoint of its potential application to determining the effective value of the scaling exponent *γ*, which would enable one to scale the dependences of the translational relaxation times τ(T,V) according to the PDS law. Based on the first argumentations presented within the framework of the isomorph theory, we decided to study the potential WU correlation in isochoric conditions. To do that, we performed additional MD simulations in the GB model in the NVT ensemble in which the WU correlation was earlier typically considered [17,18,19,29]. This approach ensured that the isochoric conditions were met at each selected volume for tested anisotropy aspect ratios. In each case of *α_r_*, we fixed three different volumes ranged representatively over the supercooled liquid state to perform the NVT MD simulations in the GB model. As a result, at all fixed volumes, we obtained perfect linear WU correlations (depicted in the insets to Figure 7), the determination errors of slope coefficients of which are less than 0.001. As expected, the obtained values of the slope coefficients *γ_WU_* for the isochoric linear WU correlations depend on the particle number density, $\rho =V^{-1}$, ranging as follows: <0.7299; 0.7874>, <0.7042; 0.7576>, <0.6803; 0.7299>, and <0.7042; 0.8333> for the examined anisotropy aspect ratios *α_r_* = 1.30, 1.35, 1.40, and 1.45, respectively. Moreover, a decrease is observed in the values of *γ_WU_* with increasing ρ resulting from a decrease in the simulation box volume. In the aforementioned ranges of *ρ*, the values of *γ_WU_* change as follows: <6.164; 6.087>, <6.217; 6.120>, <6.258; 6.110>, and <6.290; 6.065> for *α_r_* = 1.30, 1.35, 1.40, and 1.45, respectively. Hence, the sought-after effective values $\gamma_{WU}^{eff}$, which would be close to the values of *γ* followed from the density scaling criterion, might be some mean values that should be included in the value ranges of *γ_WU_* estimated from the WU correlations for the considered anisotropy aspect ratios. Taking into account the value ranges of *γ_WU_* compared with the values of the scaling exponent *γ* evaluated based on the density scaling criterion, it follows that such effective values $\gamma_{WU}^{eff}$ shown in Table 3 have to be larger than the values of *γ*. Consequently, the attempt made at scaling the dependences τ(T,V) as functions $\tau (\rho^{\gamma_{WU}^{eff}}/T)$ shows a worse quality density scaling (Figure 7) than that achieved by using the density scaling exponent *γ* (Figure 6), especially at high translational relaxation times *τ*. This is quite reasonable if we consider the aforementioned decreasing dependence, $\gamma_{WU}\left(\rho \right)$, for a given anisotropy aspect ratio *α_r_*.

Concerning the search for the effective scaling exponent $\gamma_{WU}^{eff}$ based on the WU correlations, it should be noted that the special reduced units were suggested within the framework of the isomorph theory to ensure the density scaling of molecular dynamics. The units indeed enabled the improvement of the density scaling of dynamic and thermodynamic quantities collected in the isotropic models of supercooled liquid in the limited thermodynamic range [51]. However, the isomorph theory has been declared for Newtonian and Brownian dynamics in the canonical and microcanonical ensembles, i.e., the NVT and NVE ensembles, where E denotes the total system energy. Since the isomorph theory has not been worked out for the isothermal–isobaric ensemble (NPT), it is not justified to employ the reduced units of the isomorph theory to test the density scaling properties in the simulation data collected in the NPT ensemble. The same remark is valid for the experimental data usually measured in isobaric or isothermal conditions that well correspond to the statistical ensemble defined by invariant thermodynamic variables *p* and *T*. Therefore, we neglected the reduced units of the isomorph theory in the density scaling study of the relaxation times determined herein from the NPT MD simulations.

It is worth noting that our previous investigations of the scaling exponent $\gamma_{WU}^{eff}$ performed [52] in the aforementioned RLM model also showed a discrepancy between the values of $\gamma_{WU}^{eff}$ and *γ* that actually enables one to scale the diffusion coefficient and the structural relaxation time collected from the MD simulations in the NVT ensemble. Furthermore, we have argued there that the intramolecular interactions break the isochoric linear WU correlation. Comparing our earlier MD simulation study in the RLM model with our current one conducted in the GB model, one can claim that the molecular anisotropy makes the isochoric linear WU correlation useless in the determination of the effective scaling exponent even if such a correlation is found for a molecular system in a temperature–pressure range. There are also known model systems for which the WU linear correlation is not satisfied, for instance, due to intramolecular interactions, but the density scaling of dynamic quantities has been satisfactorily validated in those models [52,53,54]. All the facts seriously undermine the WU correlation applications in the analyses of the density scaling behavior.

### 2.3. Rotational Dynamics

To investigate the rotational molecular dynamics of the supercooled liquid state in the GB model, we calculated the standard time-dependent rotational autocorrelation function *C*_2_(*t*) based on the second order Legendre polynomial *P*_2_, which is additionally described in Section 3. However, to evaluate the timescale *τ_rot_* of the rotational molecular dynamics at the same decay level of the relaxation function at which the timescale *τ* is determined for the translational molecular dynamics in the GB supercooled liquid, we assumed that a measure of *τ_rot_* is the time satisfying the equation, $C_{2}\left(t\right)=e^{-1}$.

**Figure 8:**
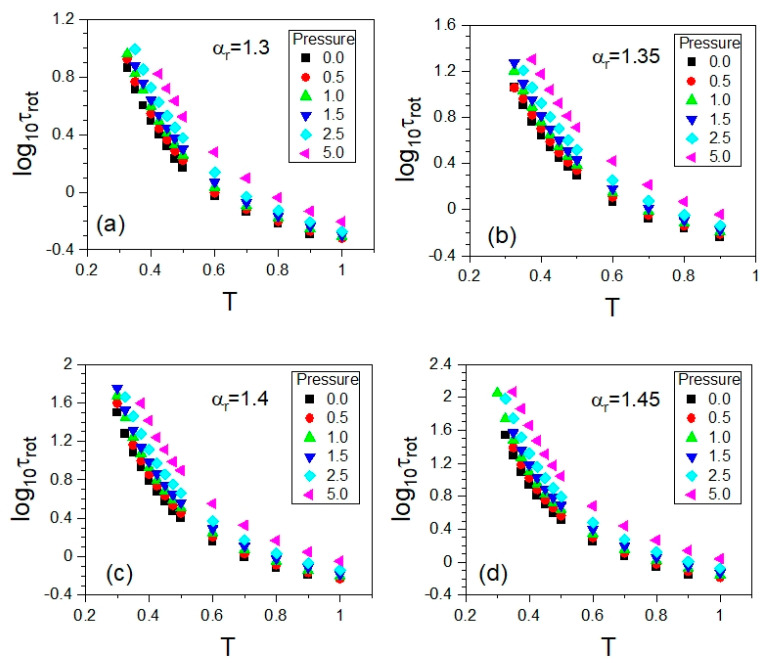
Plots of the isobaric dependences of rotational relaxation times *τ_rot_* on temperature *T* in the supercooled liquid state in the GB model, which are presented in the panels for the different anisotropy aspect ratios *α_r_*: (**a**) 1.30, (**b**) 1.35, (**c**) 1.40, and (**d**) 1.45, respectively.

The rotational autocorrelation functions *C*_2_ were calculated at the same state points (*T*,*p*) at which the ISS functions *F_s_* were computed. The same NPT MD simulation runs were exploited to evaluate both the rotational and translational time-dependent autocorrelation functions *C*_2_ and *F_s_* at a given (*T*,*p*); comparative examples are presented in Section 3. Materials and Methods. As a result, we obtained the *T*-*p* and *T*-*V* dependences of *τ_rot_*, where the average values of the particle number volume *V* were obtained from the MD simulation in the NPT ensemble at each state point (*T*,*p*). Since the NPT MD simulations were carried out along selected isobars, in an analogous way to the translational relaxation times, one can plot both isobaric dependences, $\tau_{rot}(T)$ and $\tau_{rot}(V)$. The former dependences are shown in Figure 8 by using a separate panel for each anisotropy aspect ratio, while the corresponding isobaric dependences $\tau_{rot}(V)$ are presented in Appendix A. Thus, the thermodynamic evolutions of the timescales of rotational and translational molecular dynamics were explored in the same thermodynamic range. Taking this opportunity, we also tested the potential validity of the density scaling for rotational relaxation times *τ_rot_*. 

**Figure 9:**
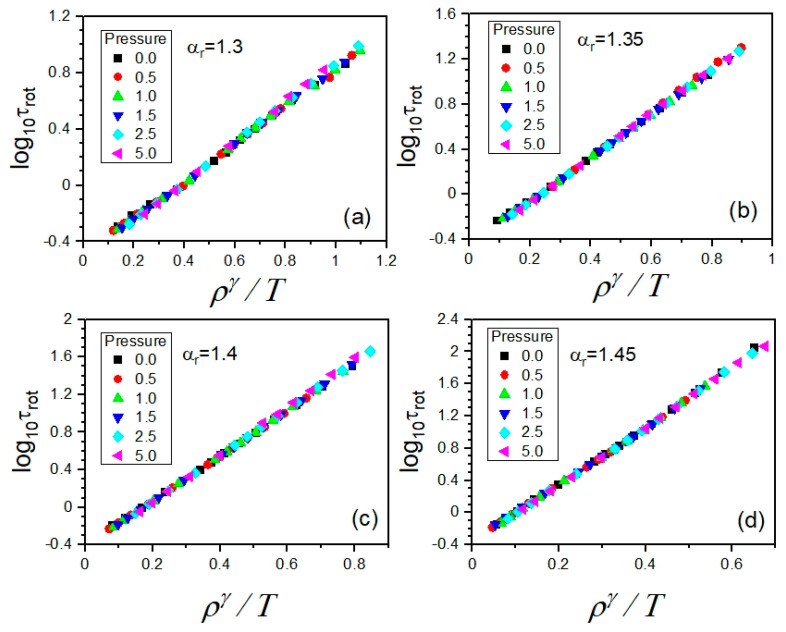
Density scaling of the isobaric dependences of rotational relaxation times *τ_rot_* for different anisotropy aspect ratios α_r_ in the supercooled liquid state in the GB model. The values of the scaling exponent *γ* are the same as those yielding the high-quality density scaling of the rotational relaxation times, which were determined from the density scaling criterion. In the separate panels, there are the cases shown for the different anisotropy aspect ratios *α_r_*: (**a**) 1.30, (**b**) 1.35, (**c**) 1.40, and (**d**) 1.45, respectively.

Since the translational and rotational motions were governed by the same GB potential in the exploited simulation model, the first attempt at scaling the dependences $\tau_{rot}(T,V)$ was made by using the values of the scaling exponent *γ*, leading to the scaling of the dependences τ(T,V). As argued in Section 2.2, the best density scaling of the latter has been achieved by exploiting the values of *γ* determined from the density scaling criterion, which are collected in Table 2. Employing these values of the scaling exponent *γ* in preparing the plots of the dependences $\tau_{rot}(T,V)$ as functions of the variable $\rho^{\gamma }/T$, we yielded a high-quality density scaling for each examined anisotropy aspect ratio, as shown in Figure 9. This finding constitutes a promising contribution to a better understanding of interrelations between different kinds of molecular motions, which is worth developing in the future. 

## 3. Materials and Methods

### 3.1. Simulations

All MD simulations were performed for 1000 ellipsoidal molecules of the same kind for a given molecular aspect ratio by using the LAMMPS package [55] within the GB potential implementation [56] available there, which relies on some earlier investigations of the GB model, e.g., [57,58]. As already mentioned, we parameterized the GB potential according to that suggested by Kapko et al. [36] All simulation runs were computed on the GPU NVIDIA Tesla V100 cards at the double-precision level and three-dimensional periodic boundary conditions suitable for the cubic simulation box. We considered only prolate spheroids in the form of the biaxial ellipsoidal molecules, the molecular shape of which is characterized by a long molecular axis and a short molecular axis perpendicular to the long one, and the examined anisotropy aspect ratios *α_r_* were determined by the quotients of the lengths of the long and short axes. All quantities used in and resulting from our MD simulations are expressed in accordance with typical LJ simulation units, and the volume *V* and the density *ρ* mean the particle number volume and the particle number density, respectively. 

Within the LAMMPS package, the commonly known leap-frog algorithm was exploited at the time step Δt=0.001 for all the simulation runs, setting the cutoff radius for interactions $r_{cut}=3.2$. The standard Nosé–Hoover thermostat and barostat were used to satisfy the thermodynamic conditions in the NPT and NVT ensembles. The barostat and thermostat relaxation times were set to 1.0 and 0.1, respectively. The periods of equilibration and subsequent well-equilibrated simulation runs depended on the state point and became longer and longer on approaching the glass transition. The equilibration runs took minimum 10^6^ time steps and the subsequent simulation runs exploited to collect the data for further analyses ranged from 10^5^ to above 10^8^ time steps. The supercooled liquid and glassy states were achieved, avoiding any crystalline ordering by cooling the isotropic liquid system, earlier simulated at a high temperature, *T* = 2.0. Functions used to monitor and analyze the molecular dynamics of the simulation systems are described in the next section.

### 3.2. Calculations

We coded most functions employed in analyses of the MD simulation results using Python programming language and its NumPy and CuPy packages based mainly on definitions of the functions presented in [59]. Only thermodynamic quantities were determined exploiting the LAMMPS package functionalities. 

To verify whether a translational ordering occurs in the system, we applied the system radial distribution function defined as an ensemble average quantity in general as follows:

(4) $$g(r)=\frac{V}{N^{2}}\langle \sum_{j=1}^{N}\sum_{l\neq j}^{N}\delta \left(r-r_{jl}\right)\rangle ,\;\mathrm{where}\;r_{jl}=r_{j}-r_{l}.$$

**Figure 10:**
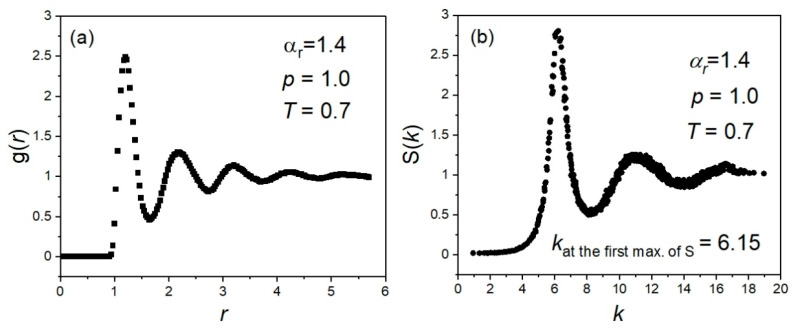
Presentation of (**a**) the radial distribution function plotted vs. the distance *r* and (**b**) the static structure factor plotted vs. the length of the wave vector *k*, selected as examples for the system of molecules of the aspect ratio $\alpha_{r}=1.4$, simulated in the GB model in the NPT ensemble at the pressure *p* = 1.0 and the temperature *T* = 0.7.

Equation (4) has been implemented in our analyses according to the algorithm described in Section 3.2 in reference [59], which provides the dependence g(r). All the obtained plots of the radial distribution function *g* on the distance *r* showed a behavior typical of disordered systems, as shown for a selected example in Figure 10a. 

To test whether an orientational ordering exists in the system, we used two functions: the order parameter *S*_2_ and the second-rank pairwise orientational correlation function g_2_. The former is defined in the following way:

(5) $$S_{2}=\langle \frac{1}{N}\sum_{j=1}^{N}P_{2}\left(e_{j}\cdot n\right)\rangle \;$$

where the brackets < > denote the ensemble average, the second order Legendre polynomial is expressed as $P_{2}(x)=1.5x^{2}-0.5$, **e***_j_* is a unit vector pointing along the long axis of the biaxial ellipsoidal *i*-th molecule, and **n** is the so-called director [59]. The director **n** has been obtained typically as the largest eigenvalue found from the diagonalization of the second-rank orientational order tensor:

$$Q_{\alpha \beta }=\frac{1}{N}\sum_{j=1}^{N}\left(\frac{3}{2}e_{j\alpha }e_{j\beta }-\frac{1}{2}\delta_{\alpha \beta }\right)\;\mathrm{where}\;\;\alpha ,\beta =x,y,z.$$

For all the exploited simulation runs, the obtained values of *S*_2_ are close to zero and did not exceed 0.06, which shows that there is no orientational order in the examined systems. This has also been confirmed by the inspection of the second-rank pairwise orientational correlation function defined as the following ensemble average quantity:

(6) $$g_{2}\left(r\right)=\langle P_{2}\left(\cos\left(\theta_{jl}\left(r\right)\right)\right)\rangle$$

where *P*_2_ is the second-order Legendre polynomial and $\theta_{jl}\left(r\right)$ denotes the angle between the long axes of the particles *j* and *l* lying in a narrow shell of center-of-mass separations $r_{jl}\approx r$ [60], which revealed very small fluctuations of decreasing amplitude, actually yielding $g_{2}\left(r\right)\approx 0$ to a very good approximation.

Thus, using the functions defined by Equations (4)–(6), we undoubtedly validated the isotropic ordering of all the simulation systems considered by us in the analyses presented herein.

As already stressed, in the study of translational motions, we focused on the movements of the centers of mass of the ellipsoidal GB particles of different anisotropy aspect ratios. For this reason, after confirming that there was no translational and orientational ordering in the investigated thermodynamic range of our bulk systems simulated in the standard cubic box, it was reasonable to evaluate the translational relaxation times *τ* based on the commonly used assumption, $F_{s}\left(\tau \right)=e^{-1}$, where the time-dependent incoherent self-scattering (ISS) function can be given by the following formula [61]:

(7) $$F_{s}(t)=\langle \frac{1}{N}\sum_{j=1}^{N}\exp\left[ik\cdot \left(r_{j}\left(t\right)-r_{j}\left(0\right)\right)\right]\rangle$$

where the brackets < > denote the ensemble average, *j* represents an *j*-th particle among *N* particles of the system, **r** indicates of the center of mass of a particle, and **k** is the wave vector taken at the location of the first maximum of the static structure factor:

(8) $$S\left(k\right)=N^{-1}\sum_{j=1}^{N}\sum_{l=1}^{N}\langle \exp\left[ik\cdot \left(r_{j}-r_{l}\right)\right]\rangle ,$$

where the wave vector $k=\left(\;2\pi /L\right)\left(n_{x},n_{y},n_{z}\right)$ is determined using the simulation box length *L* and the integer numbers *n_x_*, *n_y_*, *n_z_*. It is worth noting that the static structure factor is also a good measure of the degree of translational order in the centers of mass (p. 207, [59]). All the obtained dependences of the static structure factor *S* on the length of the wave vector, k=|k|, have revealed a behavior typical of disordered systems, as shown for a selected example in Figure 10b.

The rotational relaxation times *τ* were estimated by using the time-dependent rotational autocorrelation function *C*_2_:

(9) $$C_{2}=\langle \frac{1}{N}\sum_{j=1}^{N}P_{2}\left(e_{j}\left(0\right)\cdot e_{j}\left(t\right)\right)\rangle =\langle \frac{1}{N}\sum_{j=1}^{N}\left(\frac{3{\left(e_{j}\left(0\right)\cdot e_{j}\left(t\right)\right)}^{2}-1}{2}\right)\rangle$$

on the assumption that $C_{2}\left(\tau_{rot}\right)=e^{-1}$ to consider the same decay level of both the rotational and translational relaxation functions. We verified that the rotational relaxation times determined in this manner well corresponded with those calculated by integration of the time-dependent autocorrelation function.

**Figure 11:**
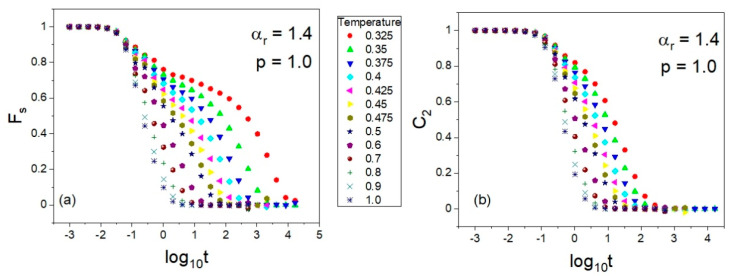
Examples of the translational (**a**) and rotational (**b**) time-dependent autocorrelation functions *C*_2_ and *F_s_* obtained respectively via Equations (7) and (9) from the NPT MD simulation data collected in the supercooled liquid state at the pressure *p* = 1.0 in the GB model of the molecules of the aspect ratio $\alpha_{r}=1.4$.

Examples of the series of the translational and rotational correlation functions *F_s_* and *C*_2_ obtained at a constant pressure (*p* = 1.0) are shown in Figure 11a and Figure 11b, respectively. 

## 4. Summary and Conclusions

For the first time, the single-component ellipsoidal Gay–Berne model has been successfully used in a simulation study of the supercooled liquid state and the glass transition at elevated pressure. Contrary to the single-component Lennard–Jones liquid model, the GB supercooled liquid is characterized by the sufficient glass formation ability in the thermodynamic range, giving the possibility of quite convenient investigations of the translational and rotational molecular dynamics near the glass transition. It seems that this advantage of the GB model over the LJ one originates from the molecular anisotropy inherent in both the ellipsoidal shapes of interacting species and the anisotropic intermolecular potential of the GB model compared to the point interacting species and the isotropic intermolecular potential of the LJ model. However, the glass-forming ability of the single-component GB model is limited to small anisotropy aspect ratios *α_r_* ranging from 1.3 to 1.5, and even the range of *α_r_* has narrowed to 1.3–1.45 to avoid entirely a liquid crystal phase ordering in the explored temperature–pressure range, 0.1≤T≤1.0 and 0≤p≤5.0, in the LJ units. In case of higher values of *α_r_*, the GB model is well suited to its typical applications, that is, to model different liquid crystal phases. One could suspect that the thermodynamic range of supercooling in the GB model might be extended by studying binary mixtures of ellipsoidal species of different *α_r_*, which is worth testing in the future.

The *pVT* data investigations have led us to identify the glassy and supercooled liquid states in the *T*-*p* domain for the assumed parametrization of the anisotropic GB model. The glass transition curves *T_g_*(*p*) detected from the volumetric data analysis for *α_r_* = 1.30, 1.35, 1.40, and 1.45 have shown a typical increasing behavior with increasing *p*, and an increase in the glass transition temperature *T_g_* with increasing *α_r_*. The latter constitutes a hypothesis that is worthy of further verification by using experimental data for glass-forming materials. 

We thoroughly explored the density scaling properties in the supercooled liquid state in the anisotropic GB model. We confirmed the validity of the density scaling of translational and rotational relaxation times expressed by some functions $\tau (\rho^{\gamma }/T)$ and $\tau_{rot}(\rho^{\gamma }/T)$ in the GB supercooled liquids of four different anisotropy aspect ratios *α_r_*, finding that the density scaling exponent *γ* is the same for *τ* and *τ_rot_* at a given *α_r_* and increases with increasing *α_r_*. We have shown that the best way to evaluate the proper value of *γ* consists in the use of the density scaling criterion. It should therefore be treated as a macroscopic parameter related to the effective short-range intermolecular potential *U_eff_* commonly suggested to comprise a dominant repulsive inverse power law term and a weak attractive background. 

In accordance with the density scaling analyses of measurement data, but in contrast to the MD simulations in simple isotropic models of supercooled liquids, the proper value of *γ* is about two times smaller than the value of parameter *γ_EoS_* found from fitting *pVT* data to a class of equations of state based on the effective intermolecular potential *U_eff_*. It can be related to a difference in the sensitivity of different quantities to the mean field effect. Such an interpretation was earlier suggested for the macroscopic quantities analyzed using experimental data [62] and subsequently proposed based on the MD simulations in the RLM model [44,45]. The latter explicitly reflects the anisotropy effect on the molecular dynamics in the rhombus-like molecular shape, which implies an anisotropy of the effective intermolecular potential that, however, results mainly from a superposition of the isotropic interactions such as the LJ and Coulomb forces. In the GB model, both the molecular shape and intermolecular potential are explicitly anisotropic. Thus, the suggested interpretation of the discrepancy between *γ* and *γ_EoS_* gains a firm confirmation in our study.

The possible support from the isomorph theory in the evaluation of the proper value of the scaling exponent *γ* has not been validated in the tested GB supercooled liquids. The values of the effective value of the scaling exponent $\gamma_{WU}^{eff}$ based on the virial–potential energy correlations have led to worse quality density scaling of translational relaxation times than those obtained using the density scaling criterion. In addition, any application of the reduced units of the isomorph theory has not been permitted to improve the density scaling, because the isomorph theory did not work for the NPT ensemble, but only for the NVT and NVE ones. In this context, it is worth noting that the isothermal-isobaric ensemble reflects the experimental thermodynamic conditions in the best way, because the vast majority of measurements are carried out at constant pressure or constant temperature. Thus, a theory that is unable to consider the NPT ensemble may be insufficient to describe comprehensively the phenomena experimentally observed. 

Our investigations of the supercooled liquid state and the glass transition in the anisotropic GB model clearly show that the anisotropic models constitute a promising alternative to the isotropic ones towards a better understanding and proper reflection of the physicochemical properties of the glass-forming materials. 

## Figures and Tables

**Table 1:** The values of the fitting parameters of Equation (3) established for all examined anisotropy aspect ratios in the supercooled liquid state in the GB model. The determination errors of the values of the EoS parameters are estimated as the standard deviation errors found from fitting all pVT simulation data to Equation (3) for a given anisotropy aspect ratio.

*α_r_*	*γ_EoS_*	*A*_0_	*A*_1_	*A*_2_	*b*_0_	*b*_1_
1.30	8.62 ± 0.18	1.2693 ± 0.0005	0.347 ± 0.004	0.297 ± 0.007	67.3 ± 0.9	2.61 ± 0.04
1.35	8.62 ± 0.17	1.3166 ± 0.0006	0.390 ± 0.005	0.317 ± 0.008	62.4 ± 0.8	2.70 ± 0.04
1.40	8.66 ± 0.17	1.3679 ± 0.0006	0.426 ± 0.005	0.358 ± 0.009	58.7 ± 0.8	2.84 ± 0.04
1.45	8.69 ± 0.17	1.4157 ± 0.0007	0.466 ± 0.006	0.388 ± 0.011	56.6 ± 0.9	2.98 ± 0.05

**Table 2:** The values of the density scaling exponent *γ* evaluated according to the density scaling criterion for all examined anisotropy aspect ratios *α_r_* as the slope coefficient of the linear isochronal correlations presented in Figure 5. The determination errors of the slope coefficient values are estimated as the standard deviation errors found from the linear regression of all the linear isochronal correlations shown in Figure 5 for a given anisotropy aspect ratio in accordance with the density scaling criterion expressed by Equation (2).

*α_r_*	*γ*
1.30	4.52 ± 0.08
1.35	4.86 ± 0.04
1.40	4.95 ± 0.08
1.45	5.09 ± 0.08

**Table 3:** The effective values of the density scaling exponent $\gamma_{WU}^{eff}$ evaluated as mean values of slope coefficients of the linear isochoric correlations presented in the insets in the panels of Figure 7 between the instantaneous values of the total system virial <W> and the total system potential energy <U> for all examined anisotropy aspect ratios *α_r_*. The determination errors of the effective values of the density scaling exponent $\gamma_{WU}^{eff}$ are the standard deviation errors of the mean value of the slope coefficient values of the isochoric linear dependences shown in the insets in the panels of Figure 7 for a given anisotropy aspect ratio.

*α_r_*	$\gamma_{WU}^{eff}$
1.30	6.13 ± 0.01
1.35	6.17 ± 0.01
1.40	6.19 ± 0.01
1.45	6.19 ± 0.01

**Figure A2:**
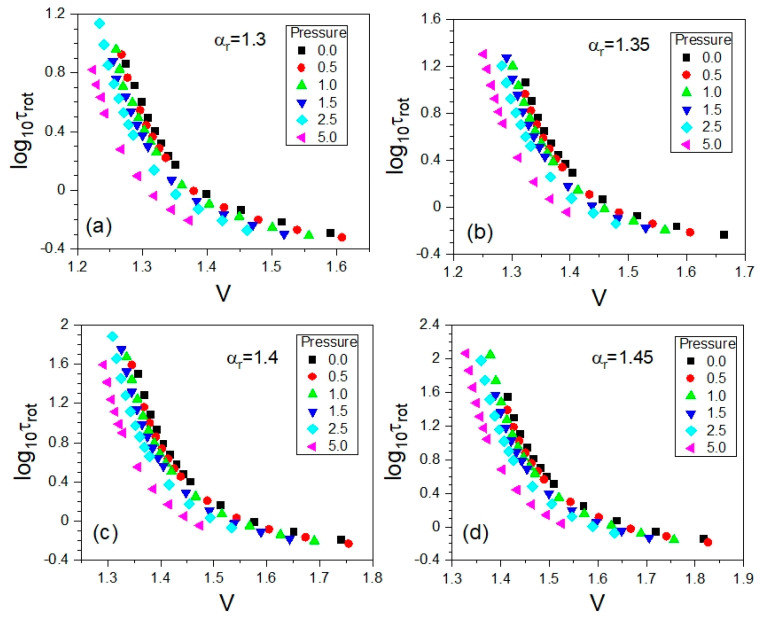
Plots of the isobaric dependences of translational relaxation times *τ* on the particle number volume *V* for different anisotropy aspect ratios *α_r_* in the supercooled liquid state in the GB model. In the separate panels, there are the cases shown for the different anisotropy aspect ratios *α_r_*: (**a**) 1.30, (**b**) 1.35, (**c**) 1.40, and (**d**) 1.45, respectively.

**Figure A3:**
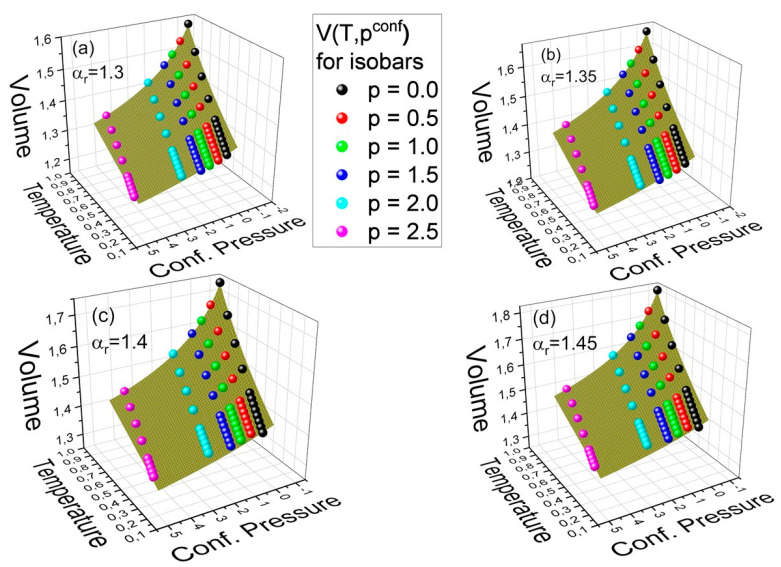
The plots of the dependences of the particle number volume *V* on temperature *T* and the configurational pressure *p^conf^* in the supercooled liquids states in the GB model. The meshed surfaces represent the fits to the EoS given by Equation (A1) with the value of its parameters collected in Table A1. In the separate panels, there are the cases shown for the different anisotropy aspect ratios *α_r_*: (**a**) 1.30, (**b**) 1.35, (**c**) 1.40, and (**d**) 1.45, respectively.

## Data Availability

The data presented in this study are available from the corresponding author upon request.

## Appendix A

In Figure 3 and Figure 8, there are plots of the temperature dependences of the logarithms of the translational and rotational relaxation times, respectively. Herein, we present plots for dependences of the same timescales on the particle number volume *V* in Figure A1 and Figure A2, respectively. The volumes were determined as the mean values directly from the simulation runs in the NPT ensemble. These volumes are also used in Figure 1 and employed in preparing all the scaling plots presented above in Figure 4, Figure 6, Figure 7 and Figure 9.

## Appendix B

In our analyses based on the equation of state, Equation (3) was exploited, because it was previously employed in many investigations of the measurement data collected for supercooled liquids. However, to complete the discussion of the scaling exponent obtained from fitting the *pVT* data as a parameter *γ_EoS_* of the equation of state, we need to consider also the equation of state, which is analogous to Equation (3), but involves the configurational pressure, $p^{conf}=\langle W\rangle /\langle V_{box}\rangle$, instead of the total system pressure *p*, where $\langle V_{box}\rangle$ is the average volume of the simulation box. This is because an isothermal precursor of Equation (3) has been derived from the total system average virial 〈W〉 on the assumption of the homogeneous potential functions that meet Euler’s theorem, which can be well interpreted in the density scaling regime, and represented by the following formula [30,31]:

(A1) $$V(T,p^{conf})=V(T,p_{0}^{conf}){\left[1+\frac{\gamma_{EOS}}{B_{T}^{conf}(p_{0}^{conf})}\left(p-p_{0}^{conf}\right)\right]}^{1/\gamma_{EOS}}$$

which can be parametrized similarly to Equation (3) with regard to temperature [32]:

$$V(T,p_{0}^{conf})={\left[\sum_{l=0}^{l=2}A_{l}^{conf}{\left(T-T_{0}\right)}^{l}\right]}^{-1},$$

$$B_{T}^{conf}(p_{0}^{conf})=b_{0}^{conf}\exp\left[-b_{1}^{conf}(T-T_{0})\right],$$

where $b_{0}^{conf}=B_{T_{0}}^{conf}(p_{0}^{conf})$, $b_{1}^{conf}=b_{1}^{conf}(p_{0}^{conf})=-{\partial \ln B_{T}^{conf}(T,p_{0}^{conf})/\partial T|}_{T=T_{0}}$, $A_{0}^{conf}=\rho^{-1}(T_{0},p_{0}^{conf})$, and $A_{l}^{conf}={(1/l!)\partial^{l}\rho^{-1}(T,p_{0}^{conf})/\partial T^{l}|}_{T=T_{0}}$ for *l* = 1, 2, are fitting parameters, and $\left(T_{0},p_{0}^{conf}\right)$ is a fixed reference state point, which is usually chosen near the glass transition at ambient pressure. Herein, we have selected $\left(T_{0},p_{0}^{conf}\right)$ at *p*_0_ = 0 and the glass transition temperatures, $T_{0}=T_{g}\left(p_{0}\right)$, established at 0.298, 0.307, 0.319, and 0.325 for the examined anisotropy aspect ratios *α_r_* = 1.30, 1.35, 1.40, and 1.45, respectively. The corresponding values of the configurational pressure at the reference state were calculated according to the formula, $p_{0}^{conf}=p_{0}-k_{B}T_{0}\rho (T_{0},p_{0})$, which is valid for MD simulations in the GB model quantified in the LJ units taking the Boltzmann constant $k_{B}=1$. The configurational pressure is not constant along an isobar determined at a constant pressure, as can be easily seen from the equation, $p^{conf}=p_{0}-k_{B}T\rho$, which interprets the configurational pressure as the difference between the total system pressure and the kinetic contribution to the total system pressure. Thus, the dependences of the particle number volume *V* on *T* and *p^conf^*, where $p^{conf}=\langle W\rangle /\langle V_{box}\rangle$, obtained from the simulation data collected in the GB model in the NPT ensemble, were fitted to Equation (A1) as a two-variable function. As a result, limiting the fitting procedure to the supercooled liquid state, we found very satisfactory fits represented by the fitting surfaces in Figure A3 with the values of the fitting parameters of Equation (A1) collected in Table A1. The values of *γ_EoS_* obtained from fitting the *p^conf^* *VT* simulation data to Equation (A1) are slightly larger than those established from fitting the *pVT* simulation date to Equation (3) and considerably larger than those evaluated from the WU correlation. This explains why the density scaling not shown herein with the scaling exponent values *γ_EoS_* listed in Table A1 is unsatisfying, similar to that shown in Figure 4, where we used the scaling exponent values *γ_EoS_* listed in Table 1.

**Table A1:** The values of the fitting parameters of Equation (A1) established for all examined anisotropy aspect ratios in the supercooled liquid state in the GB model. The determination errors of the values of the EoS parameters are estimated as the standard deviation errors found from fitting all *pVT* simulation data to Equation (A1) for a given anisotropy aspect ratio.

*α_r_*	*γ_EoS_*	*A*_0_ *^conf^*	*A*_1_ *^conf^*	*A*_2_ *^conf^*	*b*_0_ *^conf^*	*b*_1_ *^conf^*
1.30	8.92 ± 0.07	1.2693 ± 0.0002	0.349 ± 0.003	0.077 ± 0.012	62.1 ± 0.3	2.02 ±0.01
1.35	8.94 ± 0.06	1.3155 ± 0.0002	0.393 ± 0.002	0.070 ± 0.012	57.7 ± 0.2	2.07 ± 0.01
1.40	8.96 ± 0.04	1.3553 ± 0.002	0.399 ± 0.005	0.061 ± 0.010	57.2 ± 0.6	1.86 ± 0.05
1.45	9.02 ± 0.06	1.4158 ± 0.0002	0.486 ± 0.004	0.036 ± 0.017	50.9 ± 0.3	2.27 ± 0.01
